# The Association of Research Quantitative Measures With Faculty Ranks of Australian and New Zealand Dental Schools

**DOI:** 10.7759/cureus.47271

**Published:** 2023-10-18

**Authors:** Ayesha Fahim, Sadia Shakeel, Farhan Saleem, Ijaz Ur Rehman, Kashif Siddique, Habib Ahmad Qureshi, Muhammad Sohail Zafar

**Affiliations:** 1 Department of Oral Biology, The University of Lahore, Lahore, PAK; 2 Department of Public Health, Edith Cowan University, Joondalup, AUS; 3 Department of Oral Medicine, The University of Lahore, Lahore, PAK; 4 Department of Biostatistics, The University of Lahore, Lahore, PAK; 5 Department of Biomedical Sciences/Anatomy and Histology, King Faisal University, Al-Ahsa, SAU; 6 Department of Restorative Dentistry, Taibah University, Al Munawwarra, SAU; 7 School of Dentistry, University of Jordan, Amman, JOR; 8 Department of Dental Materials, Islamic International Dental College, Riphah International University, Islamabad, PAK

**Keywords:** teeth, universities, research, dentistry, faculty

## Abstract

Introduction: The scholarly productivity of a faculty member can be measured through several indicators including annual appraisals, feedback, and the number of publications per year. The present study aims to assess the association of quantitative research measures and academic ranks in Australian and New Zealand dental schools.

Methods: It was an analytical observational cross-sectional study. Full-time faculty members working in Australia and New Zealand's dental schools were discovered on official websites. Various bibliometric parameters including h-index, total number of citations, total number of publications, and maximum number of sources of a single publication were analyzed. Spearman rank correlation was used to determine the correlation between bibliometric variables and academic ranks (lecturer, assistant professor, professor). The Mann-Whitney U test was used to compare bibliometric parameters among departments (Basic and Clinical) and gender (male and female).

Results: Through the present search strategy, 207 full-time faculty members were identified, of which 12 were from New Zealand, and 195 were from Australia. Among them, 130 (62.8%) were male and 70 (33.8%) were female faculty members. There was a positive correlation of all bibliometric parameters with academic ranks (p = 0.001). There was no statistical difference between the two countries for academic parameters (p > 0.05). Male faculty members showed significantly higher academic productivity than female members in Australian dental schools (p = 0.001).

Conclusion: These bibliometric parameters and other educational parameters can be considered for determining faculty promotions. These bibliometric parameters appear to be suitable metrics for assessing research productivity, impact, and visibility.

## Introduction

Dental faculty members perform a multitude of roles in their organizations to climb up the academic ladder, including academic teaching, clinical teaching, providing patient care, involvement in administrative positions, continuing dental education, and scholarly activities [1]. Even though each dental school sets up its criteria for faculty promotion, it is usually based on some combination of academic, clinical, and research productivity [2]. Scholarly productivity can be assessed through ‘Annual appraisal forms’, exceptional feedback and other formal awards [3]. Clinical productivity is usually determined by measuring relative value units, reporting quality patient indicators and/or practice income [4].

With recent advances in digital publication, there has been a dramatic increase in scientific research. Online manuscript submission, reduced printing costs, open access to articles and rapid publication have facilitated an overwhelming amount of literature of varying quality [5]. Traditionally, the research productivity of a faculty member was measured by the number of publications, grants and international representation [6]. Although popular, these measures do not objectively assess scholarly work [7]. Several quantitative measures have emerged in recent years to overcome this shortcoming to evaluate the researcher's value in the field. Counting the total number of citations of a publication provides insight into the impact of the researcher's work. However, this method could be biased if the researcher writes just one seminal article referenced several times, and his other work remains uncited [8]. The I-10 index measures the number of articles cited 10 or more times by an author [9]. Journal websites have begun displaying the frequency of article downloads as a popularity metric. “Altmetric attention score” is provided to an article by tracking publication’s mentions on various social media platforms such as Twitter, LinkedIn, ResearchGate, Facebook, YouTube blog posts and news outlets [10]. Still, this measure does not differentiate between deliberate promotion and genuine public demand for an article [11].

The Hirsch index (h-index) was designed to combine the frequency of citations with the number of publications in a geometric progression, generating composite scores to simplify research productivity measures [12]. For instance, an author who has published five articles will have an h-index of 5 if each article has been cited at least five times. If the author has published ten articles and each has been cited at least five times, the h-index will still be 5; this is an objective measure of citations that describes the researcher's impact and, thus, the quality of his work.

Initially developed for natural sciences, the h-index has been extensively calculated to measure researcher impact in the fields of radiology, surgery, otolaryngology, pediatric dentistry and urology, where studies reveal a positive correlation between academic rank and h-index [13,14]. Another startling similarity in these studies is that research was conducted on US faculty members and thus is not generalizable. There has been an increased emphasis on the value of published scientific data in the recent years. The results of our study will shed light on the relationship of bibliometric measures and faculty academic ranks. This study assesses the association between quantitative research measures and academic ranks in Australian and New Zealand dental schools.

## Materials and methods

Study design

An analytical, observational, cross-sectional study was conducted in December 2022. Since the data were obtained from publicly available databases and patient information or healthcare details were not included in the survey, the Ethical Review Board (ERB) was not needed. Using the faculty list provided on the official websites of Australian and New Zealand dental schools, the names of all the faculty members working in Dental schools of Australia and New Zealand were acquired. The STROBE guidelines for observational studies were used to conduct and draft this study [15].

Study population

The population consisted of only full-time faculty working members appointed in Dental schools. Part-time faculty members, adjunct faculty, recently joined faculty members (in 2022), and faculty members not registered or found on the official Dental school website were omitted.

Study variables

Primary study variables-outcomes included Academic rank (Senior lecturers with postgraduate qualification/Assistant professor/Associate professor/Full professor), department (Basic sciences/Clinical sciences), and gender (Male/Female). Primary study variable-predictors were bibliometric variables of academic predictivity, which included the h-index, total number of publications, total number of citations, and maximum number of citations of a single publication. 

Data collection

A total of eight dental schools were identified. For each school, the names of faculty members from the departmental webpage were queried. Each faculty member's academic activity was analyzed using documentation techniques. For this, surnames and names of faculty members were searched in SciELO, Google Scholar, LinkedIn, and SCOPUS databases, and their number of articles was recorded. Their bibliometric parameters were recorded using a citation database for peer-reviewed literature (Scopus, Reed Elsevier, London, UK).

Data analysis

Data were entered into SPSS version 23.0 (IBM Corp., Armonk, NY). Descriptive statistics were carried out to provide an overview of the population variables. Spearman rank correlation was done to assess the correlation between academic faculty rank and quantitative research measures. The least significant differences procedure was used for multiple comparisons testing. Receiver-operator characteristic (ROC) curves were generated, and logistic regression analyses were computed. A p-value of less than 0.05 is considered statistically significant.

## Results

Through the present search strategy, 207 full-time faculty members were identified, of which 12 were from New Zealand, and 195 were from Australia. Among them, 130 (62.8%) were male and 70 (33.8%) were female faculty members. A total number of full professors identified from New Zealand and Australian dental schools were six and 44, respectively; a number of associate professors were three and 44, respectively, whereas the total number of lecturers were three and 99, respectively. We did not find any assistant professor working in New Zealand or Australian dental school during data collection. The detailed demographics are presented in Table 1. Total number of publications was higher for Australia as compared to New Zealand. The mean number of publications was higher for New Zealand dental (51±46) colleges than for Australia (44±65). Although the mean h-index, total number of citations and maximum number of citations was higher for New Zealand dental colleges, but when compared with Australian dental colleges, no significant difference was obtained. 

**Table 1:** Characteristics of study sample N = number, % = percentage, SD = standard deviation

	New Zealand, N (%)	Australia, N (%)	Total, N (%)
Number	12	195	207
Gender			
Male	10 (83.3)	127 (65.1)	130 (62.8)
Female	2 (16.7)	68 (34.9)	70 (33.8)
Academic Rank			
Professor	6 (50)	44 (22.6)	50 (24.1)
Associate Professor	3 (25)	44 (22.6)	47 (22.7)
Assistant Professor	0 (0)	0 (0)	0
Lecturer	3 (25)	99 (50.8)	102 (49.2)
Department			
Clinical	12 (100)	154 (79)	166 (80.1)
Basic	0 (0)	40 (20.5)	40 (19.3)
Total Publications	616	7,519	
	Mean±SD	Mean±SD	P-value
Mean Number of Publications	51±46	44±65	-
H-Index	16.17±13.35	11.57±11.30	0.214
Total number of citations	1808±2283	1321±4527	0.190
Maximum number of Citations	222±329	209±989	0.249

Table 2 presents the mean h-index score for different designations in New Zealand and Australian Dental Colleges. The mean h-index score was compared within and between countries across different designations. For New Zealand, the mean h-index score did not show significant variation across designations. However, higher h-index values can be seen for higher rank, which gradually decreases with lower designation/rank. For Australia, a significant difference was seen in the mean h-index score between designations. The highest mean h-index score was for professors, followed by associate professors, and lecturers had the lowest mean h-index score.

**Table 2:** Comparison of bibliometric measures in relation to designation Note: *(Kruskal Wallis Test) & **: Mann-Whitney U Test N = number, SD = standard deviation, Multiple Comparison test Lecture vs. Associate Professor:0.013*, Lecture vs. Professor: <0.001*, Associate Professor vs. Professor: <0.001*

Rank of Faculty member	Number (N)	H-Index in New Zealand (mean ± SD)	H-Index in Australis (mean ± SD)	P-value** (Between Designation)
Professor	50	24.00±14.18	23.60±12.58	0.831
Associate Professor	47	12.66±5.03	10.91±9.42	0.425
Lecturer	102	4.00±5.29	5.62±4.98	0.406
p-value*	-	0.127	0.001*	-

**Figure 1:**
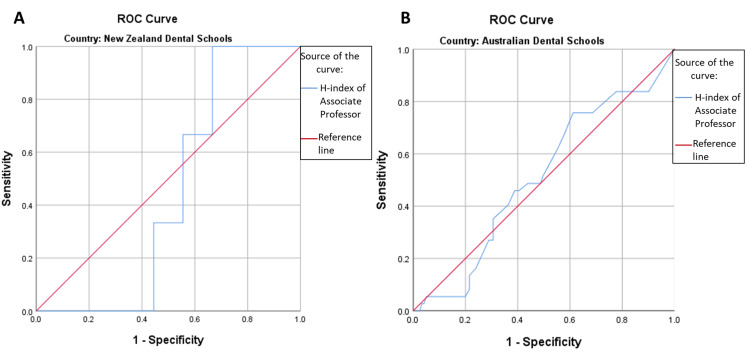
Receiver-operator characteristic curves for associate professor (h-index) ROC = Receiver-operator characteristic (A) ROC curve for faculty members of New Zealand Dental Schools showing area under the curve: 0.444, p-value=0.782, h-index cut point=9.00 (Sensitivity=0.667 & 1-Specifcity=0.667) (B) ROC curve for faculty members of Australian Dental Schools showing area under the curve: 0.509, p-value=0.866, h-index cut point=2.50 (Sensitivity=0.838 & 1-Specifcity=0.777)

For the New Zealand Dental College Associate Professor, the cut-off value for h-index was 9.00, and for the Australian Dental Colleges Associate Professor, the cut-off value for h-index was 2.50. The area under the curve for both countries is low, i.e., AUC (New Zealand=0.444, p-value=0.782) and AUC (Australia=0.509, p-value=0.866) (Figures 1A, 1B).

**Figure 2:**
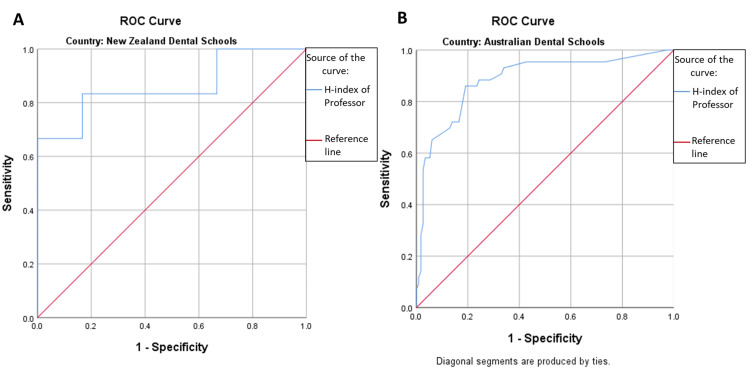
Receiver-operator characteristic curves for professor (h-index) ROC = Receiver-operator characteristic (A) ROC curve for faculty members of New Zealand Dental Schools showing area under the curve: 0.861, p-value=0.037, h-index cut point=14.00 (Sensitivity=0.833 & 1-Specificity=0.167) (B) ROC curve for faculty members of Australian Dental Schools showing area under the curve: 0.884, p-value=0.000, h-index cut point=12.50 (Sensitivity=0.860 & 1-Specificity=0.191)

For New Zealand Dental College, the Professor cut-off value for h-index was 14.00 and for Australian Dental Colleges, the Professor cut-off value for h-index was 12.50. The area under the curve for both countries is excellent, i.e., AUC (New Zealand=0.861 p-value=0.037) and AUC (Australia=0.884, p-value<0.001) (Figures 2A, 2B).

H-index was compared in relation to departments as well as gender in both countries. All study participants from New Zealand were from clinical departments. The mean h-index score for these participants was 16.16±13.3. No significant difference between the basic and clinical departments was seen in the h-index for study participants from Australia. However, the h-index was higher for study participants from the Basic Sciences Department. For New Zealand dental college, no significant difference was seen in the h-index score between male and female study participants. However, for Australian dental colleges, the h-index score was significantly higher for males than females, i.e., p-value<0.001 (Table 3).

**Table 3:** Comparison of bibliometric measures in relation to department Note: *statistically significant at p≤0.05 N = number, SD = standard deviation

	Department			Gender		
New Zealand	Basic	Clinical	P-value	Male	Female	P-value
Faculty members (N)	N=0	N=12		N=10	N=2	
H-index (mean ± SD)	-	16.16±13.3	-	17.40±14.378	10±2.82	0.606
Number of publications (mean ± SD)	-	51.33±46.90	-	56.40±50.07	26±9.89	0.606
Total no. of Citations (mean ± SD)	-	1808.41±2283.96	-	2098.70±2410.48	357±176.77	0.606
Max. no. of Citations (mean ± SD)	-	222.33±329.16	-	255.80±353.23	55±41.01	0.606
Australia	Basic	Clinical	P-value	Male	Female	P-value
Faculty members (N)	N=36	N=129		N=107	N=58	
H-index (mean ± SD)	14.44±12.63	10.76±10.80	0.095	13.76±12.02	7.51±8.50	<0.001*
Number of publications (mean ± SD)	56.45±69.34	41.45±64.62	0.143	56.32±72.66	23.38±43.69	<0.001*
Total no. of Citations (mean ± SD)	2970.47±9038.26	860.89±1718.18	0.152	1766.11±5506.18	500.32±1224.70	0.001*
Max. no. of Citations (mean ± SD)	597.69±2063.32	100.69±168.59	0.094	290.50±1221.60	62.27±126.36	0.001*

Table 4 indicates the correlation between bibliometric measures and academic ranks. There is a strong positive correlation in both Australian and New Zealand dental schools. Overall, there is a strong correlation between the h-index, the total number of publications and the total number of citations (r = >0.6-0.7) and academic ranks, and a moderate correlation between academic ranks with the maximum number of citations (r = 0.4-0.5).

**Table 4:** Correlation between bibliometric measures and academic rank N = number, r = correlation coefficient Note: r = >0.6-0.7 (Strong Correlation), 0.4-0.5 (Moderate Correlation) *Statistically significant at p≤0.05

Bibliometric Measure	New Zealand (N=12)		Australia (N=195)		Total (N=207)	
-	r	P-value	r	P-value	r	P-value
H-Index	0.706	<0.001*	0.620	<0.001*	0.634	<0.001*
Total Number of Publications	0.741	<0.001*	0.585	<0.001*	0.602	<0.001*
Total number of citations	0.649	<0.001*	0.614	<0.001*	0.623	<0.001*
Max number of citations	0.649	<0.001*	0.535	<0.001*	0.551	<0.001*

## Discussion

The present study assessed the association of quantitative research measures and academic ranks in Australian and New Zealand dental schools. The cohort of full-time faculty members working in dental schools from their official websites were identified. Data were found from one New Zealand and seven Australian dental schools. The bibliometric parameters assessed were the number of publications, the h-index, the total number of citations, and the maximum number of citations. There was no statistical difference between the bibliometric parameters of the two countries. Although the two countries have separate dental councils and accreditation bodies for medical and dental universities, the teaching and learning methodologies, their system of promotion, and registration to practice are quite similar, which is why any dentist registered by one country can work in the other country through their Trans-Tasman Mutual Recognition (TTMR) Act established in 1997 [16]. Even overseas faculty member who works in Australia or New Zealand are recognized on the same parameters and have similar experiences, as evidenced by previous studies [17-20]. Thus, it can be expected that faculty research performance and publication trends are similar in both countries. For future studies, a comparison of bibliometric parameters between different continents, especially with Asian countries should be included.

In this research, the bibliometric parameters of basic and clinical faculty members of the two countries were compared. There were no significant differences between the type of specialty. Previous data could not be found to compare the results. This could be because schools usually do not differentiate between their basic sciences faculty and clinical sciences faculty. Thus, a team of faculty members is involved in teaching various modules in a clinically designed setting [21]. This, however, paves the way for future research where biomedical sciences experts and an increase in medical and dental school faculty. The current study revealed a statistically significant difference between genders, where male faculty members have higher academic productivity when compared to female faculty members. This result is in line with several other studies where gender disparities exist among faculty members. Significantly decreased representation of female doctors in higher positions has been identified in the United States, Canada, England [22], Japan [23], China [24], etc. As women climb the academic ladder, these disparities drastically increase [25]. No single explanation for this disparity was found. The future qualitative analysis could determine the reason and possible ways to counter this disparity.

These parameters were associated with the academic rank of faculty members. All bibliometric parameters had a positive correlation with academic ranks. These results are in line with previous studies conducted on faculty or oral and maxillofacial surgery [26] and pediatric dentistry [2]. However, the correlation values are slightly different in these studies, where only the h-index is strongly correlated with academic ranks, while the rest of the parameters are moderately correlated (r = 0.4-0.5). Similar correlations can be found in other studies conducted on hand surgeons [27] and craniofacial surgeons [28]. The difference in correlation could be explained by the fact that a few years ago, only the h-index was considered as a measure of academic productivity [29], but now other measures are also given equal importance, and thus, faculty are encouraged to produce a larger number of papers with a high number of citations. The results also reflect the promotion criteria of dental schools. The earlier criteria of higher degree and high work experience are no longer entertained for faculty promotions. Universities are stressing their faculty members to produce high-quality publications, which in turn gets them high university rankings [30].

This study had a few limitations. Firstly, only the data provided on the university's official websites were considered, thus, a few newly recruited faculty members could be missed. Differentiation between tenure-tracked and non-tenure-tracked faculty members could not be done since this information was not available for every faculty member. With increasing diversity in dental schools, further studies should be conducted to compare bibliometric parameters of inter-continent faculty members to establish differences in faculty promotion and academic performance criteria in different regions of the world. While advancement in academic institutions should be based upon multiple factors like education, years of experience, teaching, administrative services, community services, clinical productivity, etc., the bibliometric parameters hold the utmost importance in the professional advancement of dental faculty.

## Conclusions

This study uncovered a positive correlation between academic ranks and several bibliometric parameters, including the h-index, total publications, total citations, and maximum citations, within dental schools in both Australia and New Zealand. Notably, there were no significant distinctions in these parameters between the two countries. However, it is worth highlighting that male faculty members exhibited significantly higher academic productivity in Australian dental schools. In conclusion, these findings suggest that bibliometric parameters play a crucial role in assessing the quality of scholarly work and should be taken into account, if not as the sole criterion, in the promotion of faculty members within dental schools.
